# Mapping Canadian Data Assets to Generate Real-World Evidence: Lessons Learned from Canadian Real-World Evidence for Value of Cancer Drugs (CanREValue) Collaboration’s RWE Data Working Group

**DOI:** 10.3390/curroncol29030165

**Published:** 2022-03-17

**Authors:** Wei Fang Dai, Claire de Oliveira, Scott Blommaert, Reka E. Pataky, David Tran, Zeb Aurangzeb, Cynthia Kendell, Chris Folkins, Chandy Somayaji, Jeff Dowden, Winson Cheung, Erin Strumpf, Jaclyn M. Beca, Carol McClure, Robin Urquhart, James Ted McDonald, Riaz Alvi, Donna Turner, Stuart Peacock, Avram Denburg, Rebecca E. Mercer, Caroline Muñoz, Ambica Parmar, Mina Tadrous, Pam Takhar, Kelvin K. W. Chan, on behalf of the CanREValue Collaboration

**Affiliations:** 1 Temerty Faculty of Medicine, University of Toronto, Toronto, ON M5S 1A8, Canada; weifang.dai@mail.utoronto.ca; 2 Canadian Centre for Applied Research in Cancer Control, Canada; rpataky@bccrc.ca (R.E.P.); jaclyn.beca@ontariohealth.ca (J.M.B.); speacock@bccrc.ca (S.P.); rebecca.mercer@ontariohealth.ca (R.E.M.); 3 Centre for Health Economics and Hull York Medical School, University of York, York YO10 5DD, UK; claire.deoliveira@camh.ca; 4 ICES, Toronto, ON M4N 3M5, Canada; 5 Sunnybrook Health Sciences Centre, Toronto, ON M4N 3M5, Canada; smblommaert@uwaterloo.ca (S.B.); ambika.parmar@sunnybrook.ca (A.P.); 6 BC Cancer, Vancouver, BC V5Z 1G1, Canada; 7 Saskatchewan Cancer Agency, Regina SK S4W 0G3, Canada; david.tran@saskcancer.ca (D.T.); riaz.alvi@saskcancer.ca (R.A.); 8 CancerCare Manitoba, Winnipeg, MB R3E 0V9, Canada; zaurangzeb@cancercare.mb.ca (Z.A.); dturner9@cancercare.mb.ca (D.T.); 9 Department of Community Health Sciences, University of Manitoba, Winnipeg, MB R3E 0W3, Canada; 10 Department of Surgery, Dalhousie University, Halifax, NS B3H 2Y9, Canada; cynthia.kendell@ccns.nshealth.ca (C.K.); robin.urquhart@nshealth.ca (R.U.); 11 New Brunswick Institute for Research, Data and Training, University of New Brunswick, Fredericton, NB E3C 1N8, Canada; chris.folkins@unb.ca (C.F.); csomayaj@unb.ca (C.S.); tedmcdon@unb.ca (J.T.M.); 12 Cancer Care Program, Eastern Health, St. John’s, NL A1B 3V6, Canada; jeff.dowden@easternhealth.ca; 13 Department of Oncology, University of Calgary, Calgary, AB T2N 4N2, Canada; winson.cheung@albertahealthservices.ca; 14 Department of Epidemiology, Biostatistics, and Occupational Health, McGill University, Montreal, QC H3A 2T7, Canada; erin.strumpf@mcgill.ca; 15 Ontario Health (CCO), Toronto, ON M5G 2L7, Canada; caroline.munoz@ontariohealth.ca (C.M.); pam.takhar@ontariohealth.ca (P.T.); 16 Prince Edward Island Cancer Registry, Government of Prince Edward Island, Charlottetown, PE C1A 9L2, Canada; carol.mcclure@peihealth.ca; 17 Division of Haematology/Oncology, The Hospital for Sick Children, Toronto, ON M5G 1X8, Canada; avram.denburg@sickkids.ca; 18 Women’s College Hospital, Toronto, ON M5S 1B2, Canada; mina.tadrous@wchospital.ca; 19 Leslie Dan Faculty of Pharmacy, University of Toronto, Toronto, ON M53 3M2, Canada

**Keywords:** real-world data, oncology, population-based

## Abstract

Canadian provinces routinely collect patient-level data for administrative purposes. These real-world data (RWD) can be used to generate real-world evidence (RWE) to inform clinical care and healthcare policy. The CanREValue Collaboration is developing a framework for the use of RWE in cancer drug funding decisions. A Data Working Group (WG) was established to identify data assets across Canada for generating RWE of oncology drugs. The mapping exercise was conducted using an iterative scan with informant surveys and teleconference. Data experts from ten provinces convened for a total of three teleconferences and two in-person meetings from March 2018 to September 2019. Following each meeting, surveys were developed and shared with the data experts which focused on identifying databases and data elements, as well as a feasibility assessment of conducting RWE studies using existing data elements and resources. Survey responses were compiled into an interim data report, which was used for public stakeholder consultation. The feedback from the public consultation was used to update the interim data report. We found that databases required to conduct real-world studies are often held by multiple different data custodians. Ninety-seven databases were identified across Canada. Provinces held on average 9 distinct databases (range: 8–11). An Essential RWD Table was compiled that contains data elements that are necessary, at a minimal, to conduct an RWE study. An Expanded RWD Table that contains a more comprehensive list of potentially relevant data elements was also compiled and the availabilities of these data elements were mapped. While most provinces have data on patient demographics (e.g., age, sex) and cancer-related variables (e.g., morphology, topography), the availability and linkability of data on cancer treatment, clinical characteristics (e.g., morphology and topography), and drug costs vary among provinces. Based on current resources, data availability, and access processes, data experts in most provinces noted that more than 12 months would be required to complete an RWE study. The CanREValue Collaboration’s Data WG identified key data holdings, access considerations, as well as gaps in oncology treatment-specific data. This data catalogue can be used to facilitate future oncology-specific RWE analyses across Canada.

## 1. Introduction

In recent years, real-world evidence (RWE) has gained increasing interest from decision makers with its potential to inform and support regulatory reviews, health technology assessments (HTAs), reimbursement decisions and price negotiations for novel therapies [1,2,3,4]. Traditionally, health technology assessment reviews have relied on evidence from randomized clinical trials (RCTs) to assess a drug’s clinical benefit [5,6]. With increasing real-world studies examining post-market outcomes of drugs in clinical practice, there is growing evidence to suggest that effectiveness in the real-world may differ from efficacy observed in RCTs [7,8,9,10,11]. While RCTs are the gold standard for establishing a treatment’s efficacy, clinical trials may not be representative of all patients from the general population who will receive the drug in clinical practice due to highly selective trial eligibility criteria [12]. This efficacy–effectiveness gap can be particularly troubling for decision makers evaluating novel anticancer therapies because of the rapidly evolving therapeutic space and high drug prices. In particular, previous studies in the literature have demonstrated that cost-effectiveness estimates derived from economic models using clinical trial data were often underestimation of the incremental cost-effectiveness ratios generated using real-world data [13,14,15]. As such, RWE, generated by the analysis of real-world data (RWD), can provide useful information that can inform decision makers when reassessing drug funding decisions as part of life-cycle health technology management.

RWD has been defined as data collected in a non-clinical trial setting, including data collected from electronic health records, disease registries, personal health devices, and administrative databases [12,16]. RWD have also been defined as data collected after RCTs, regulatory approvals, HTAs, reimbursement decisions or following price negotiations [12]. Since the majority of RWD is collected routinely through clinical practice or as part of the administrative claims process, it can be relatively more accessible compared to other data sources and relatively inexpensive compared to standard clinical trials, especially for jurisdictions with existing data infrastructure [12,17,18]. Consistent with patient-centered health care, the RWD collected can be used to develop many different types of information, including prevalence and incidence of disease, effectiveness and safety of treatments, as well as quality of life and patient-reported outcomes associated with treatments [16,18,19,20,21,22,23,24,25,26,27,28,29,30,31]. Stakeholders, including clinicians, researchers, and decision makers, have suggested these types of information can be useful for post-funding reassessment for cancer drugs [16,32,33]. The insights gained from analysis of RWD can inform routine clinical practice by clinicians, recommendations by HTA agencies, and price negotiations and reimbursement decisions by decision makers.

In Canada, the majority of health care is publicly funded by provincial/territorial governments [34]. Despite being a publicly funded healthcare system, there are geographical variations in cancer incidence across provinces as shown by the Canadian Cancer Society, suggestive of differences in risk factors, diagnostic practices, and data collection [35]. Publicly funded cancer treatments are routinely administered and reimbursed by the provinces either through the Ministry/Department of Health or the provincial cancer agencies/programs [36]. Data collection aligns with this funding structure, wherein different governments across Canada collect real-world, population-based administrative data on health system resource utilization for their jurisdictions, including claims data on funded cancer drugs. In addition to federal and provincial/territorial governments, the Canadian Institute for Health Information (CIHI), a federally chartered, independent, not-for-profit organization, also collects and holds pan-Canadian databases on comprehensive health care data provided by each province [37]. In 2018, CIHI developed the pan-Canadian Minimal Oncology Dataset (pCMOD) report, which is a set of data standards and guidelines, with aims to harmonize the collection of oncology drug data in alignment with national and provincial/territorial interests [38]. Despite the significant efforts by government entities and third-party organizations to harmonize data collection, a recent qualitative study of key stakeholders across Canada on the perspective of RWD noted significant concerns regarding the siloed nature of data assets in the current system [33]. Another study also noted that the varying data access, data governance, and data availability across provinces are barriers to use of RWD for drug funding studies [39]. Notwithstanding the challenges to using RWD, there is a paucity of effort to map and catalogue the data elements that currently exist in each province that can be used for real-world studies in oncology.

The Canadian Real-world Evidence for Value in Cancer (CanREValue) Collaboration was established in 2017 with the aim to develop a framework for incorporating RWE into cancer drug funding decisions [40,41,42]. As part of the CanREValue Collaboration, five working groups (WGs) were established, including the CanREValue Data WG [40]. The CanREValue Data WG was established with the aim to explore and map the existing population-based administrative healthcare databases across Canadian provinces. The CanREValue Data WG also identified a list of data elements necessary for conducting real-world studies in oncology and explored the availability of these data elements within the existing databases. This paper will outline the main findings from the CanREValue Data WG’s efforts to map existing administrative databases and data elements for conducting real-world analysis in oncology.

## 2. Approach

### 2.1. CanREValue Data Working Group

The Data WG was formed as a part of the CanREValue Collaboration and consists of 20 data experts and researchers across all 10 Canadian provinces. The objective of the Data WG was to map the databases and data elements that were available in each province which can be used to conduct cancer-specific RWE studies. From March 2018 to September 2019, the Data WG members convened for three teleconferences and two in-person meetings to iteratively identify and map the potential types of databases and data elements needed for conducting real-world retrospective administrative database studies in cancer. Following the meetings, the CanREValue Collaboration core research team developed surveys that were shared with the provincial experts for completion. Since data elements to conduct real-world studies were contained in cancer-specific and non-cancer-specific databases, both types of databases were considered in the mapping exercise. The surveys specifically aimed to explore population-based administrative databases that collect and maintain data on publicly funded health care services, as the current focus of CanREValue Collaboration centers around population-based RWE studies to inform funding decisions around publicly funded cancer drugs.

### 2.2. Surveys on Provincial Data Assets

Surveys on the data elements and databases required for conducting real-world studies were created by the CanREValue Collaboration core research team based on a previous real-world study conducted in Ontario, Saskatchewan, and British Columbia [13,43]. The first section of the survey focused on identifying databases containing relevant types of information (e.g., cancer registry data, hospitalization data, etc.), with questions including database name and custodian of the database. The second section of the survey focused on identifying data elements that are required for conducting cancer-specific real-world studies. The data elements chosen for this mapping exercise were selected during the teleconference discussions based on experiences with the feasibility of identifying these data elements from previous RWE studies conducted by the data experts. The data experts were also asked to identify the database that contains each data element, assess the availability and linkability of the data elements, and identify any limitations in coverage and/or completeness of the data element over time. The availability and linkability of each data element were categorized as (i) data available and linkable, (ii) data available and linkable with caveats, (iii) data availability and linkability to be determined after conducting RWE analysis, and (iv) data not available or linkable. The final section of the survey asked each provincial data expert to assess the feasibility of conducting an RWE study for intravenous and oral drugs based on the availability and linkability of each of the variables of interest. Data experts were asked to estimate, based on their previous experience, the time it would take for cohort creation and evaluation of each type of outcome as (i) 3–6 months, (ii) 6–12 months, and (iii) more than 12 months.

### 2.3. Stakeholder Consultation

After collecting the survey responses from the provincial experts, an interim data report was developed that contained information on the available data assets from the mapping exercise. A public stakeholder consultation on the interim data report was initiated from 13 November 2019 to 13 December 2019. The interim data report was publicly posted on the CanREValue Collaboration website (https://cc-arcc.ca/canrevalue/ (13 November 2019)) and was electronically sent to the CanREValue Collaboration mailing list, as well as on the social media account. Public feedback on the interim report was consolidated into a document and the relevant changes were incorporated into the updated interim Data Report. Along with the revised data report, the response document was published online on the CanREValue website on 21 April 2020.

## 3. Results

### 3.1. Databases for RWE Studies 

Across Canada, 97 databases were identified in this exercise. The data experts identified an average of 9 databases (range 8–11) in each province that contained data elements relevant for cancer-specific RWE analysis (Table 1). For all provinces, the Ministries/Departments of Health (MoH/DoH) maintains databases on publicly funded health services that are administered through provincial health insurance plans or health authorities within their jurisdiction. Most provincial MoH/DoH work with CIHI to capture standardized hospitalization data through the Discharge Abstract Database (DAD) and ambulatory care services (including emergency department visits) through the National Ambulatory Care Reporting System (NACRS). Québec is the only province that does not fully report to the DAD, while Ontario and Alberta are the only provinces that fully report to the NACRS. In other provinces, the services administered by MoH/DoH include both cancer and non-cancer treatments while in other provinces/territories, specific care is delegated to specialized agencies. For example, in some provinces, such as Ontario, Saskatchewan, British Columbia, Manitoba, Nova Scotia, and Newfoundland and Labrador, cancer treatments/funding are administered through provincial agencies/programs and, thus, detailed treatment data may be collected by the agency/programs on behalf of the MoH/DoH. In such circumstances, data may be shared between the two organizations or may require data sharing/linking for the purpose of health system planning and administration. Since the databases required to conduct RWE studies may be held across multiple data custodians, this can create barriers for timely data access and linkage. In some provinces/territories, there are third-party organizations (e.g., ICES (formerly known as the Institute for Clinical Evaluative Sciences) in Ontario and Health Data Nova Scotia (HDNS)) that are authorized to access and link provincial demographic and health-related databases for research and evaluation.

### 3.2. Variables Required for Conducting RWE

Variables that are necessary to conduct real-world comparative analysis were categorized into three essential components: (1) variables for cohort creation; (2) variables on baseline demographic and clinical characteristics; and (3) variables on outcomes of interest.

The first component of a real-world study is to build an appropriate study cohort that can answer the research question. Variables to define the disease of interest such as cancer diagnosis codes (ICD-O-3 morphology, topography, behavior code), stage, and date of diagnosis were considered necessary for cohort selection. Variables on receipt of treatment, including a drug identifier, date of treatment, and dose administered were also considered relevant for identifying the eligible patient cohort and conducting analysis. Further, given that specific drugs may be used for more than one setting, data elements defining treatment indication, line of therapy and/or intent of treatment were also considered relevant.

The second component of an RWE study includes demographic and clinical characteristics for describing the cohort and balancing differences between treatment groups to ensure comparability. These variables included age, sex, neighborhood income quintile, region/rurality, comorbidity, performance status, and prior treatment exposures (systemic therapy, radiotherapy, and cancer-directed surgery). Concurrent or subsequent treatments (systemic therapy, radiotherapy, and cancer-directed surgery) were also included as relevant clinical characteristics to consider.

The third component of an RWE study includes the outcomes. Five key types of outcomes were identified including clinical effectiveness, safety, cost-effectiveness, budget impact, and patient-reported outcomes (Table 2). Within each type of outcome, there are distinct endpoints that can be studied. For example, endpoints within the clinical effectiveness outcome category include overall survival and other time-to-event endpoints (treatment discontinuation or progression-free survival). An initial assessment of some specific endpoints for each outcome type is listed in Table 2. The data elements required to generate these endpoints are also outlined in Table 2.

### 3.3. Mapping Real-World Data Elements in Provinces

Building upon the three essential components of a real-world study, the WG created The Essential Cancer RWD Table (Table 3), a list of data elements that are minimally necessary for conducting real-world studies in oncology. Each data element is also indicated for whether it is used for cohort creation, baseline/clinical characteristics, or outcome. For the outcome component, we designed the data element as relevant for real-world survival, real-world safety, real-world cost, or real-world budget impact. Some data elements may be needed for all three components of the RWE study such as participant ID, while some data elements may only be required for one component of the RWE study, such as cost of the drug, which is only required for real-world comparative cost-effectiveness. Since some of the variables listed in Table 2 are composite variables, such as comorbidity, multiple data elements in The Essential Cancer RWD Table are required to generate these composite variables.

The Expanded Cancer RWD Table presented in Table 4 includes a more comprehensive list of data elements, including those variables identified in The Essential Cancer RWD table in Table 3. The availability of these additional data elements within the Expanded Cancer RWD would enhance the real-world analysis but may not be routinely collected in each province. Variables that are relevant only to a specific disease or drug or are not routinely reported to population-based databases are not included in this list. While most provinces have data on patient demographics (e.g., age, sex) and cancer diagnosis related variables (e.g., morphology, topography), the availability and linkability of data on cancer treatment, clinical characteristics, and drug costs varies among provinces.

### 3.4. Resource and Capacity Assessment

The capabilities of each province to perform RWE analysis were assessed, considering currently available data holdings and resources such as dedicated personnel and funding (Table 5). Analysis capabilities were assessed separately according to the outcomes to be analyzed (based on those outlined in Table 2) as well as the route of administration of the study drug(s) (IV vs. oral). As shown in Table 5, a province’s capability to perform RWE analysis differs according to the outcomes of interest being measured, the province’s current data holdings and infrastructure, and the route of administration of the study drugs being evaluated. Many provinces estimated that they could not complete an RWE study for cancer drugs within 12 months with their current resources.

### 3.5. Stakeholder Consultation

In the public consultation with the interim data report, responses were received from stakeholders of pharmaceutical companies, industry consultancies, non-profit organizations, and patient groups. The call for feedback prompted respondents to identify additional relevant data elements that had not been listed in the report. Some data elements noted by stakeholders include race/ethnicity, physical activity, smoking, and alcohol, which are important risk factors for cancer and are useful to collect at a population level to implement preventative health policies. Other data elements such as progression, biomarker status, and overall response rate are important endpoints to understand cancer treatment and disease trajectory. While these data elements may be relevant for real-world analysis, many are not systematically collected within publicly owned population-based databases currently. It is worth noting that some of these data elements may be documented in patient charts, which can be harnessed using advanced methods such as artificial intelligence or machine learning methods. A full list of these stakeholder-identified data elements can be found in the Supplementary Table S2. While the focus of the CanREValue Data WG was on population-based administrative databases, respondents were also prompted to identify privately/academically held databases that could be used for RWE. Many respondents suggested additional Canadian or international databases, such as disease site-specific databases (e.g., the Canadian Melanoma Research Network), pediatric oncology databases (e.g., Pediatric Oncology Group of Ontario Networked Information System, POGONIS), and private databases (e.g., IQVIA and RxDynamics). These databases were compiled and shared with the public for researchers interested in conducting RWE research using privately/academically held databases (Supplementary Table S3). In the updated interim data report, the Data WG members also conducted a comparison of the identified data elements to the pan-Canadian Minimal Oncology Dataset (pCMOD) as suggested by respondents to understand the concordance between the necessary data elements [38] (Supplementary Table S1).

## 4. Discussion

The CanREValue Collaboration’s Data WG conducted a descriptive study to map the existing real-world population-level administrative data assets across Canadian provinces. An inventory of key data custodians and databases maintaining RWD throughout each province was compiled. Two data asset inventories were identified, one containing a list of minimally necessary data elements, and another containing an expanded list of relevant data elements for conducting cancer-specific RWE studies. In addition to the different availability of data elements for conducting real-world studies, the current capacity and capability within each province to perform real-world analysis also vary significantly. The majority of provinces/territories do not have the capacity to conduct RWE analyses within 12 months based on current resourcing, but most could complete an RWE analysis within 3 to 12 months if dedicated funding and personnel were available.

In Canada, there is growing interest in RWD. In 2018, CIHI published the pCMOD report that compiled a list of standard data elements that should be collected across the provinces for RWE generation [38]. Many of the data elements listed in the pCMOD were explored by the CanREValue Data WG, with some notable exceptions including data elements on the health care facility where the drug was received and prescriber information. In future iterations of the CanREValue data report, these data elements can be explored. Health Canada has also started several projects focused on the integration of RWE in drug regulatory decisions and the generation of RWE [2,46,47]. In a recent report published by Health Canada, several principles regarding the generation of decision-grade RWE were outlined including protocols around retrospective and prospective data collection [2]. The findings from this mapping exercise conducted by CanREValue Data WG can enhance previous work by CIHI and Health Canada. By exploring the existing availabilities of these data elements in each province, existing gaps within the data infrastructure that may benefit from future dedicated investments are also identified.

Our study aligns with international interests for developing RWE. The minimal dataset developed by Minimal Common Oncology Data Elements (mCODE) in the United States was created to standardize interoperability between electronic health record systems. mCODE includes data elements such as a genomics markers and laboratory results [48] that were not included in our report as they are not routinely collected in provincial administrative datasets. There have also been efforts to evaluate RWD holdings throughout Europe. The RWD holdings for most of the 160 cancer registries across EU countries have not been mapped; however, major differences in data quality are believed to exist between countries [49,50]. The minimal dataset recommended by the European Medicines Agency aligns with the minimal dataset presented in this report and includes many of the same data elements [49]. The European Network of Cancer Registries (ENCR) has also recommended essential and optional datasets specifically for tumor-based cancers. The Essential and Expanded datasets in this report are generalizable for most types of cancer but are still aligned with many of the data elements recommended by the ENCR. The ENCR’s optional dataset contains the patient’s occupation and risk for developing cancer, which was identified in our stakeholder consultation as an additional data element to be explored (Supplementary Table S3).

This work was a first step to understanding pan-Canadian data assets across all ten provinces, but there are few limitations. First, our work did not include databases from the territories or federal drug plans. Future work will be needed to explore the data assets held in these jurisdictions. Second, the reported assessment of completeness and quality of the data elements is based on a high-level review by the data experts in the WG. We anticipate that our knowledge of the data elements will be enhanced as we conduct a pilot real-world demonstration project that is currently underway. Based on our learnings, we may iteratively update the data report in the future. Finally, it is likely that some provinces may be limited in their access to databases and data elements that may not be routinely used for research purposes. As already identified by the prior qualitative study, there still remain silos within the data access process [33]. Forsea et al. proposed that an increase in stakeholder participation, increased political support from patient advocacy groups and health professionals, and the harmonization of datasets could improve RWD holdings across Europe [51].

Notwithstanding the limitations, our study is the first initiative to catalogue existing population-based databases and real-world data elements that can be used to conduct studies in oncology for the purpose of informing drug funding in Canada. Building upon insights and recommendations from previous studies, we partnered with provincial data experts to map out the existing assets and gaps of the current Canadian data infrastructure. This catalogue of existing data assets is an essential and practical first step towards the vision of a pan-Canadian interprovincial data platform that can generate RWE to inform cancer drug funding decisions. Future work can be carried out to explore the differences in population-level data elements between provinces and to address these gaps. Lastly, our work highlights the importance and success of collaboration between different jurisdictions and stakeholders and may serve as an example to promote future efforts to advance data infrastructure and access.

## 5. Conclusions

In conclusion, the CanREValue Collaboration’s Data WG conducted a mapping exercise that identified a data asset inventory of databases and data elements that are required to perform real-world analysis. Moreover, the CanREValue Data WG also provided an estimate of the capacity and capability required to complete real-world analysis based on existing circumstances and future ideal state. Using findings from this process, the CanREValue Collaboration has initiated a pan-Canadian multi-provincial real-world study. Following the real-world study, the Data WG will update the tables of data elements based on our first-hand experience accessing and analyzing the data. With continued efforts from the CanREValue Collaboration, RWE could be used to better assess and refine cancer drug funding across Canada, thus supporting cancer drug sustainability and value for money.

## Figures and Tables

**Table 1:** Summary of databases in provinces.

Province	Data Custodian	Databases
British Columbia (BC)	BC Cancer	BC Systemic Therapy Program
		BCC Radiotherapy Database
		BCC Surgery Database
		BCC CAIS Scheduling Database
		BC Cancer Registry
	BC Ministry of Health, accessed via Population Data BC (PopData)	National Ambulatory Care Reporting System
		Discharge Abstract Database
		Medical Services Plan (MSP) Payment Information File
		PharmaNet (including PharmaCare)
		Home and Community Care
		Vital Statistics Deaths file
Alberta (AB)	Alberta Health Services	Alberta Cancer Registry
		Pharmaceutical Information Network
		Alberta Blue Cross Claims
		Population Registry
		Practitioner Claims
		Diagnostic Imaging
		National Ambulatory Care Reporting System
		Discharge Abstract Database
		Alberta Continuing Care Information System
	Service Alberta	Vital Statistics—Death Registry
		Vital Statistics—Birth Registry
Saskatchewan (SK)	Saskatchewan Cancer Agency	Saskatchewan Cancer Registry
		Oncology Pharmacy Database
		Clinical Management System: ARIA MO (Medical Oncology)
		Clinical Management System: ARIA RO (Radiation Oncology)
	Saskatchewan Ministry of Health	Physician Claims-MSB
		Discharge Abstract Database
		National Ambulatory Care Reporting System
		Continuing Care Reporting System
Manitoba (MB)	CancerCare Manitoba (CCMB)	Clinical Management System: ARIA MO (Medical Oncology)
		Population Oncology Drug Program database
		Manitoba Cancer Registry and Treatment
		Clinical Management System: ARIA RO (Radiation Oncology)
	Manitoba Health, Seniors and Active Living	Manitoba Health Insurance Registry
		Medical Claims
		Drug Program Information Network
		Discharge Abstract Database
		National Ambulatory Care Reporting System
	Manitoba Vital Statistics	Vital Statistics Mortality
Ontario (ON)	Cancer Care Ontario (CCO)	New Drug Funding Program
		Activity Level Reporting System
		Ontario Cancer Registry
		Symptom Management
	Ministry of Health	Registered Persons Database
		Ontario Health Insurance Plan
		Ontario Drug Benefit
		Home Care Database
		Discharge Abstract Database
		National Ambulatory Care Reporting System
		Continuing Care Reporting System
Québec (QB)	Régie de l’assurance maladie du Québec (RAMQ)	Fichier d’inscription des personnes assurées
		Services rémunérés à l’acte
		Fichier d’admissibilité au régime général d’assurance médicaments
		Maintenance et exploitation des données pour l’étude de la clientèle hospitalière
		Banque de données communes des urgences
		Système d’information sur la clientèle et les services des CSSS-mission CLSC
		Services pharmaceutiques
	Ministère de la Santé et des Services sociaux du Québec	Performance hospitalière
		Fichier des tumeurs
		Registre québécois du cancer
	Institut de la statistique du Québec	Fichier des décès
New Brunswick (NB)	NB Department of Health (accessed via NB Institute for Research, Data and Training)	Cancer Treatment Access Repository–Radiation Therapy
		Citizen Database
		NB Cancer Data
		NB Physician Billing
		NB Prescription Drug Programs
		Drug Information System
		Discharge Abstract Data
	NB Department of Social Development (accessed via NB Institute for Research, Data and Training)	Long-Term Care Data
	Vitalité Health Network (accessed via NB Institute for Research, Data and Training)	IV Oncology (Vitalité)
Nova Scotia (NS)	Nova Scotia Health	Nova Scotia Cancer Registry
		Oncology Patient Information System
		Hospital pharmacy databases
	IWK	Breast Imaging System
	Nova Scotia Department of Health and Wellness (accessible via Health Data Nova Scotia)	Discharge Abstracts Database
		Seniors’ Pharmacare
		Medical Services Insurance Physician Billings
		Nova Scotia Drug Information System (community pharmacy data)
Newfoundland and Labrador (NL)	NL Cancer Care Program (Accessed through Eastern Health)	Clinical Management System: ARIA MO (Medical Oncology)
		Clinical Management System: ARIA RO (Radiation Oncology)
		NL Cancer Registry
		Provincial Systemic Therapy Database
		Oncology Patient Information System
	NL Centre for Health Information	Pharmacy Network
		NL Prescription Drug Program Database
		Medical Care Plan Billing
	Eastern Health, Central Health, Western Health and Labrador Grenfell Health	Meditech
	CIHI	Discharge Abstract Database
Prince Edward Island (PEI)	PEI Cancer Treatment Center	PEI Cancer Registry
		ARIA
	Health PEI	Clinical Information System
		Discharge Abstract Database
		National Ambulatory Care Reporting System
	Department of Health and Wellness	Drug Information System
		Claims Processing System—Medicare
		PharmaCare

ECOG = Eastern Cooperative Oncology Group, Note: ARIA is a registered trademark of Varian Medical Systems, Inc.

**Table 2:** Outcomes of interest for conducting a real-world study.

Outcome of Interest	Endpoints	Data Elements
Clinical Effectiveness	Overall survival Other time-to-event endpoints	First date of treatment, date of death or event of interest (e.g., treatment discontinuation, progression, etc.), study end date, date of last contact with the healthcare system.
Safety and Toxicity	Hospitalizations Emergency department visits	Date of visit, reason for visit/visit disposition.
Cost-Effectiveness	Cost data Incremental cost-effectiveness ratio Incremental net-benefit regression	Costs of systemic therapy drugs, costs of outpatient prescription drugs, costs of radiotherapy, costs of surgery, cost of hospitalizations, physician billing, costs of home care, costs of palliative care, costs of continuing and long-term care, costs of other ambulatory care, quality of life, willingness-to-pay threshold.
Budget Impact	Budget over X time period	Cost of drug, number of patients per year, height of patient, weight of patient, dose per patient, dose per cycle, cycles per patient, treatment duration, market size (number of patients eligible for treatment), and market share (% of patients receiving drug of interest).
Patient Reported Outcomes	Quality-of Life Measures Disease specific symptom measures	Patient reported outcomes, patient reported experience measure, date of collection.

**Table 3:** Essential Cancer RWD Table.

Data Element	Description	Database	Purpose		
			Cohort	Covariate	Outcome
Provincial Patient ID	Unique patient identifier	All databases used	Y	Y	Y—Linkage
Diagnosis Topography code	ICD-O-3 Code from ICD to identify the part of the body affected by disease or the site of origin of the neoplasm	Cancer Registry	Y		
Diagnosis Morphology code	ICD-O-3 Code from the morphology section of the ICD to identify the microscopic structure of cells, tissues, and organs	Cancer Registry	Y		
Date of diagnosis	Diagnosis date—the date of first diagnosis of the primary site of cancer	Cancer Registry	Y	Y	
Drug Identifier—Drug name/code/regimen/DIN	Drug name, regimen, or DIN (Health Canada identifier) to identify study drugs, prior and subsequent treatments	Treatment/claims	Y	Y	
Treatment date	Date of treatment for particular drug—IV medication	Treatment/claims	Y	Y	Y—Survival
Treatment dose given	Dose given to patient for IV medication	Treatment/claims			Y—Budget Impact
Drug (IV)-total cost	Cost of dose administered to patient (unless calculated from total amount administered and unit cost)	Treatment/claims			Y—Costs
Dispensing date	Dispensing date for particular drug—oral medication	Treatment/claims (outpatient prescriptions)	Y	Y	
Doses dispensed—Days supplied	Estimated number of days supplied or amounts dispensed—oral medication	Treatment/claims (outpatient prescriptions)			Y—Budget Impact
Drug (oral)—total cost	Total cost of dispensed drug (unless calculated from total amount dispensed and unit cost)	Treatment/claims (outpatient prescriptions)			Y—Costs
Sex/Gender	Patient sex	Population Registry	Y	Y	
Date of birth	Date of birth	Population Registry	Y	Y	
Postal code	To determine categories of neighborhood income quintile, rurality	Population Registry, Census data		Y	
Date of death	Date of death	Population Registry, Vital Statistics	Y		Y—Survival
Surgical Intervention code CCP/CCI Code	The CIHI CCP/CCI procedure code describing the procedure administered to the patient	CIHI-DAD		Y	Y—Safety
Surgical resection date	Date of surgical intervention associated with CCP/CCI codes	CIHI-DAD	Y	Y	Y—Safety
Discharge date of hospitalization	Discharge date	CIHI-DAD		Y	Y—Safety
Date of admission of hospitalization	Date of admission to acute care	CIHI-DAD	Y	Y	Y—Safety
Visit disposition code	Status of the patient upon leaving the hospital	CIHI-DAD	Y	Y	Y—Safety
Main problem	ICD diagnosis code and type (most-responsible diagnosis)	CIHI-DAD	Y	Y	Y—Safety
Hospitalization/SDS-RIW	Resource intensity weight (RIW) to calculate cost	CIHI-DAD/NACRS			Y—Costs
Hospitalization/SDS—Cost per Weighted Case	Cost per weighted case	CIHI-DAD			Y—Costs
Physician Billing	Physician billing code (or amount paid)	Physician billings database			Y—Costs
Physician Service date	Date of physician visit	Physician billings database			Y—Costs
Radiation Use	Identifies patients who received radiation	Radiation database		Y	Y—Costs
Radiation-Intent	The intention of radiation treatment as determined by the radiation oncologist	Radiation database			Y—Costs
Radiation-visit date	The patient’s visit date	Radiation database	Y	Y	Y—Costs

ICD = International Classification of Disease.

**Table 4:** Expanded Cancer RWD Table.

Category	Variables	Description	BC	AB	SK	MB	ON	QC	NB	NS	NL	PEI
Cohort Creation: Identify disease of interests	Topography	ICD-O-3 Code from International Classification of Diseases to identify the part of the body affected by disease or the site of origin of the neoplasm						?				
	Morphology	ICD-O-3 Code from the morphology section of the International Classification of Diseases to identify the microscopic structure of cells, tissues, and organs						?				
	Behavior	Reportable histological behavior—the 5th digit of reported histology, based on reported site						?				
	Date of diagnosis	Diagnosis date—the date of first diagnosis of the primary site of cancer										
Cohort Creation: Identify treatment of interest	Drug Identifier—IV	Identifies IV drug received by patient				✓			?	?		
	Drug Identifier—Oral	Identifies oral drug received by patient					✓			?		
	Treatment Indication	Identifies specific indication for use			?	✓	✓	?	?	?	✓	
	Intent of treatment	Adjuvant, curative, or palliative	✓	✓	✓	✓	✓		?	?	✓	✓
	Line of therapy	Line of therapy such as first-line setting	✓	✓	?	✓	✓		?	?		✓
	Date of treatment administration	Date of treatment for particular drug—IV medication					✓		?	?		
	Dispensing date	Dispensing date for particular drug-oral medication					✓	✓		?		
Demographic and Clinical Characteristics	Provincial Patient Identifier	Unique patient identifier										
	Sex	Patient Sex										
	Date of Birth	Date of birth										
	Age at first treatment	Age at first treatment is derived from date of birth and date of treatment										
	Rural/Urban residence	Use postal code to identify urban or rural residence										
	Neighborhood Income Quintiles	Determined using the PFFC macro and postal code										
	Regional Health Authority	Health authority regions (if applicable)										N/A
	Charlson’s Score	Co-morbidity measure derived from hospitalizations dates and reasons for admission						?	✓		✓	?
	Adjusted Clinical Groups (ACG)	Co-morbidity measure using the John Hopkin’s ACG system and derived from hospitalization dates, reasons for admission, physician visits, and ED visits	?						✓			
	ECOG-Performance Status	Performance Status			✓	✓	✓			✓	✓	
	Palliative Performance Status	Performance Status			✓		✓			✓	✓	
	Radiation Use	Identifies patients who received radiation										
	Radiation—Dose/minutes per fraction	The dose of radiation delivered		?								
	Radiation—Intent	The intent of radiation treatment as determined by the radiation oncologist at the time of booking the planning/treatment visit. (e.g., adjuvant, curative)		?					✓		?	
	Radiation—visit date	Patient’s visit date for radiation treatment		?								
	Surgical resection code	The CIHI CCP/CCI procedure code describing the procedure administered to the patient										
	Surgical resection date	Date of surgical intervention associated with CCP/CCI codes										
Clinical Effectiveness	Date of Death	Date of death										
	Date of last contact	Variable derived from dates of healthcare service utilization (e.g., discharge date, date of last treatment)										
Safety and Toxicity	ED Visit—Date of registration [44]	Date of registration to emergency department	?		✓	✓		?	?	?		
	ED Visit—Main Problem [44]	Type of separation from the ambulatory care service	?		✓	✓		?	?	?		✓
	ED Visit—Visit disposition code [44]	Most clinically significant diagnosis, condition, problem or circumstance	?		✓	✓		?		?		✓
	Hospital Visit—Date of admission [45]	Date of admission to inpatient										
	Hospital Visit—Diagnosis codes or procedure codes [45]	Status of the patient upon leaving the hospital										
	Hospital Visit—Discharge disposition [45]	ICD diagnosis code and type (most-responsible diagnosis)										
Cost-effectiveness	Drug (IV)—total cost	Cost of dose administered to patient (unless calculated from total amount administered and unit cost)		?						?		
	Drug—reimbursed cost	Total cost of drug to a drug program, if different from total cost (i.e., if patient pays co-pay)		?					✓	?		?
	Drug (oral)—total cost	Total cost of dispensed drug (unless calculated from total amount dispensed and unit cost)		?						?		
	Drug—Dispensing fees	Total cost of drug dispensing fee to a drug program		?		✓				?		
	Drug—Compounding fee	Total cost of drug compounding fee to a drug program		?					?	?		
	Physician fee—Billing code	Billing codes for physician service	✓	?						?		
	Physician fee—Amount paid	Amount paid for physician service	✓	?						?		
	Outpatient laboratory and imaging services—Billing code	Billing codes for service	✓	?						?	?	
	Outpatient laboratory and imaging services—Amount paid	Amount paid for service	✓	?						?	?	
	ED cost/resource intensity weight	Resource intensity weight (RIW) for Comprehensive Ambulatory Classification System case mix grouping of the visit. Cost of visit calculated by multiplying the patient visit’s RIW by the cost per weighted case for the jurisdiction and year	✓	?						?	?	?
	Hospitalization cost/resource intensity weight	RIW (see above) for hospital admission case mix group grouping for the visit to calculate cost of hospitalization		?						?		
	Home Care	Cost associated with home care		?						?		
	Complex continuing care	Cost of complex continuing care		?	✓					?		?
Budget Impact	Doses dispensed—Days supplied	Estimated number of days supplied or amounts dispensed—oral medication					✓	✓	✓	?		
	Treatment dose given	Dose given to patient for IV medication		✓	✓	✓	✓		?	?	✓	
	Body Surface area	Patient’s body surface area at treatment		✓	✓	✓	✓			?	✓	
	Height	Patient’s height at treatment		✓	✓	✓	✓			?	✓	✓
	Weight	Patient’s weight at treatment		✓	✓	✓	✓			?	✓	✓
Patient reported outcomes	Edmonton Symptom Assessment Score	Patient Reported Outcomes		✓	✓	✓				?	✓	

Note: While some variables listed in the table can be captured by one data element (e.g., sex), other variables are derived from multiple data elements (e.g., age at first treatment requires both birth date and date of first treatment). Details of each variable are listed in the description column. Legend: Green color = data available and linkable; Yellow color with check mark = data available and linkable with caveats; Yellow color with question mark = data availability and linkability to be determined after conducting RWE analysis; Red color = data not available or linkable. ICD-O-3 = International Classification of Disease for Oncology Third version. IV = Intravenous; ACG = Adjusted Clinical Group. ED = Emergency Department. ECOG = Eastern Cooperative Oncology Group; CIHI = Canadian Institute for Health Information. CCI = Canadian Classification of Health Interventions. CCP = Canadian Classification of Diagnostic, Therapeutic, and Surgical Procedures.

**Table 5:** Capability assessment for conducting a population-based comparative analysis on intravenous and oral cancer drugs.

Outcomes	BC	AB	SK	MB	ON	QC	NB	NS	NL	PEI
Intravenous Drugs										
Effectiveness (survival)										
Safety and Toxicity										
Budget Impact (public payer’s perspective)										
Cost-Effectiveness Analysis										
Patient reported outcomes, quality of life										
Oral Drugs										
Effectiveness (survival)										
Safety and Toxicity										
Budget Impact (public payer’s perspective)										
Cost-Effectiveness Analysis										
Patient reported outcomes, quality of life										

Legend: Green color = analysis can be completed; Yellow color = analysis can be completed with caveats; Red color = analysis cannot be completed.

## Data Availability

Not applicable.

## Supplementary Materials

The following supporting information can be downloaded at: https://www.mdpi.com/article/10.3390/curroncol29030165/s1, Table S1: Comparison between pan-Canadian Minimal Oncology Dataset (pCMOD) and CanREValue Interim Data; Table S2: Additional real-world data elements requiring future exploration; Table S3: Potential private/academic databases for RWE analysis; Table S4: Survey on databases and data elements; Table S5: Survey on capacity assessment; Table S6: Glossary.
