# Supplementary material for: Mapping Canadian Data Assets to Generate Real-World Evidence: Lessons Learned from Canadian Real-World Evidence for Value of Cancer Drugs (CanREValue) Collaboration’s RWE Data Working Group
Source: Curr Oncol. 2022 Mar 17;29(3):2046–63. doi: 10.3390/curroncol29030165 (PMC8947246; doi:10.3390/curroncol29030165)
Supplement: Supplementary file 1 [file curroncol-29-00165-s001.zip › curroncol-1587074-supplementary.pdf]

Supplementary Table S1: Comparison between pan-Canadian Minimal Oncology Dataset (pCMOD) and CanREValue Interim Data

Category	Data Element from pCMOD	Status	Available in CanREValue Data Report	Notes
Health Service Event	Service Date	M	Yes	The date on which the prescription was dispensed
Organization Information (health care facility where the drug was received)	Organization ID	M	No	Will explore in future reports
	Organization Province	M	No	N/A – each province maintains their own datasets;
	Organization Postal Code	M	No	Will explore in future reports
Prescriber Information	Prescriber ID	O	No	Will explore in future reports
	Prescriber Specialty	O	No	Will explore in future reports
	Prescriber Province	O	Yes	N/A – each province maintains their own datasets;
	Prescriber Postal Code	O	No	Will explore in future reports
Client/patient information	Client/Patient ID	M	Yes	
	Client/Patient Province	M	No	N/A – each province maintains their own datasets;
	Client/Patient Postal Code	M	Yes	
	Client/Patient Gender	M	Yes	
	Client/Patient Date of Birth	M	Yes	
	Client/Patient Height	O	Yes	
	Client/Patient Weight	O	Yes	
	Client/Patient Body Surface Area	O	Yes	
	Body Surface Formula	C	No	N/A – This is a formula and not a variable;
Disease information	Diagnosis Code	M	Yes	
	Topography Code	M	Yes	
	Morphology Code	M	Yes	
	Topography/Morphology Code Version	M	No	Will explore in future reports
	Staging	M	Yes	
	Date of Initial Diagnosis	M	Yes	
Drug Information	Drug product ID / Name	M	Yes	
	Drug Product Strength	C	No	Will explore in future reports
	Drug Product dosage form	C	Yes	
	Regimen/Treatment Plan	O	Yes	
	Quantity Dispensed	M	Yes	
	Measurement Unit	O	No	Will explore in future reports
	Days supply	O	Yes	
	Route of Administration	O	Yes	
	Drug Cost	O	Yes	

Status: M = Mandatory; O = Optional; C = Conditional;

Supplementary Table S2: Additional real-world data elements requiring future exploration

Type	Variable
Demographic	Race/Ethnicity
	Immigration status
Tumor characteristics	Site of metastasis
	Number of metastatic sites
	Node (+/-)
	Tumor mutational burden
	Recurrence vs de novo diagnosis
	Date of progression
	Criteria for evaluating progression
	Date of response
	Response status
	Criteria for assessing response
Biomarker status	Drug and disease biomarkers status<br>(e.g. HR+, HER2+, TNBC, EGFR, ALK, ROS1, NTRK, CEA, CA19-9, CA-125)
	Biomarker assay used
	Date of test
	Date of results
Lab test	Lab test<br>(E.g. Lymphocyte count, Platelet count)
PROMS, PREMS, and QoL	Quality of life (QoL)<br>(e.g. European Organization Research and Treatment of Cancer Quality of Life Questionnaire-C30; EuroQol-5D-5L, Patient Reported Functional Status, Generalized Anxiety Disorder-7, Patient Health Questionnaire-9, Brief Pain Inventory, Chronic Fatigue Syndrome)
	Patient-reported outcome measures (PROMS)
	Patient-reported experience measures (PREMS)
Healthcare Utilization	Treatment chair time
	Nursing time
	Pharmacy time to prepare IV dose
	Advance care planning
Cancer Risk Factors/Confounders	Smoking
	Alcohol
	Sun Exposure
	Diet
	Physical Activity
	Sleep
	Stress
	Chemical exposure
	Occupational Exposure
	Genetic Changes
	Infectious disease
	Radiation
	Family History

Social Determinants of Health	Income and social status
	Employment and working conditions
	Education and literacy
	Burden of disease on productivity
	Childhood experiences
	Physical environments
	Social supports and coping skills
	Healthy behaviors
Outcome Measures	Event-free survival (EFS)
	Disease-free survival (DFS)
	Invasive-disease free survival (iDFS)
	Progression-free Survival (PFS)
	Progression-free Survival 2 (PFS2)
	Minimal residual disease (MRD)
	Pathological complete response (pCR)
	Time to next treatment (TTNT)
	Treatment-free interval (TFI)
	Overall response rate (ORR) and associated strata (stringent Complete Response, Complete Response, Very Good Partial Response, Partial Response, Stable Disease)

Supplementary Table S3: Potential private/academic databases for RWE analysis

- **Disease site specific database:**
  - Canadian Melanoma Research Network: healthie™
  - Pan-Canadian Lung Cancer Observational Study (PALEOS)
  - Uveal Melanoma Registry
  - Canadian Prostate Cancer Biomarker Network (BPCBN)
  - The Alberta Prostate Cancer Research Institute Initiative (APCaRI): Alberta Prostate Registry
  - The Myeloma Canada Research Network (MCRN) Canadian Multiple Myeloma Database
  - Canadian Bladder Cancer Information System (CBCIS)
  - Canadian Kidney Cancer Information System (CKCIS)
  - GlansLook Lung Cancer Database (Alberta)
  - McPeak-Sirois Breast Metastases Registry (Montreal Region)
  - Enhanced Pancreatic Cancer Profiling For Individualized Care (EPPIC)
- **Pediatric oncology database:**
  - Pediatric Oncology Group of Ontario Networked Information System (POGONIS)
  - Cancer in Young People in Canada (CYP-C) databases
- **IQVIA Databases: Private Drug Claims and RxDynamics**
- **The Canadian Personalized Healthcare Innovation Network (C-PHIN)'s databases**
- **Palliative care databases owned by the Alberta Health Service Palliative Care Zonal program leaders**
- **Patient Support Programs Database (PSPs)**
- **Private insurance disability registries**
- **O2 Oncology Outcomes program databases**
- **Programme de Gestion Thérapeutique des Médicaments databases**
- **Personalize my Treatment (PMT) Registry**
- **International registries:**
  - Flatiron Electronic Health Record Database (US)
  - The National Lung Cancer Registry (Sweden)
  - CRISP register (Germany)

Note: The databases were identified through Stakeholder consultation

Supplementary Table S4: Survey on databases and data elements

Category	Variables	Description	Database Name	Notes
Cohort Creation: Identify disease of interests	Topography			
	Morphology			
	Behaviour			
	Date of diagnosis			
Cohort Creation: Identify treatment of interest	Drug Identifier – IV			
	Drug Identifier – Oral			
	Treatment Indication			
	Intent of treatment			
	Line of therapy			
	Date of treatment administration			
	Dispensing date			
Demographic and Clinical Characteristics	Provincial Patient Identifier			

Category	Variables	Description	Database Name	Notes
	Sex			
	Date of Birth			
	Age at first treatment			
	Rural/Urban residence			
	Neighbourhood Income Quintiles			
	Regional Health Authority			
	Charlson's Score			
	Adjusted Clinical Groups(ACG)			
	ECOG-Performance Status			
	Palliative Performance Status			
	Radiation Use			
	Radiation – Dose/minutes per fraction			
	Radiation – Intent			
	Radiation – visit date			
	Surgical resection code			
	Surgical resection date			
Clinical Effectiveness	Date of Death			
	Date of last contact			
Safety & Toxicity	ED Visit - Date of registration[26]			
	ED Visit - Main Problem[26]			
	ED Visit - Visit disposition code[26]			
	Hospital Visit - Date of admission[27]			
	Hospital Visit - Diagnosis codes or procedure codes[27]			
	Hospital Visit - Discharge disposition[27]			
Cost-effectiveness	Drug (IV) – total cost			
	Drug – reimbursed cost			

Category	Variables	Description	Database Name	Notes
	Drug (oral) – total cost			
	Drug – Dispensing fees			
	Drug – Compounding fee			
	Physician fee – Billing code			
	Physician fee – Amount paid			
	Outpatient laboratory and imaging services – Billing code			
	Outpatient laboratory and imaging services – Amount paid			
	ED cost/resource intensity weight			
	Hospitalization cost/resource intensity weight			
	Home Care			
	Complex continuing care			
Budget Impact	Doses dispensed – Days supplied			
	Treatment dose given			
	Body Surface area			
	Height			
	Weight			
Patient reported outcomes	Edmonton Symptom Assessment Score			

Supplementary Table S5: Survey on capacity assessment

Intravenous Drug

Analysis	BC	AB	SK	MN	ON	QB	NS	NB	NFL	PEI
Cohort Creation										
Effectiveness (Survival)										
Safety & Toxicity										
Budget Impact										
Cost Effectiveness Analysis										
Patient reported outcomes										

Oral Drug

Analysis	BC	AB	SK	MN	ON	QB	NS	NB	NFL	PEI
Cohort Creation										
Effectiveness (Survival)										
Safety & Toxicity										
Budget Impact										
Cost Effectiveness Analysis										
Patient reported outcomes										

Supplementary Table S6: Glossary

AB = Alberta
ACG = Adjusted Clinical Group
BC = British Columbia
CanREValue = Canadian Real-World Evidence for Value of Cancer Drugs
CCI = Canadian Classification of Health Interventions.
CCMB = CancerCare Manitoba
CCO = Cancer Care Ontario
CCP = Canadian Classification of Diagnostic, Therapeutic, and Surgical Procedures;
CIHI = Canadian Institute for Health Information
DAD = Discharge Abstract Database
DIN = Drug Identifier Number
DoH = Departments of Health
ECOG = Eastern Cooperative Oncology Group
ED = Emergency Department.
ENCR = European Network of Cancer Registries
HDNS = Health Data Nova Scotia
HTA = Health Technology Assessment
ICD-O-3 = International Classification of Disease for Oncology Third version.
IV = Intravenous
MB = Manitoba
mCODE = Minimal Common Oncology Data Elements
MoH = Ministries of Health
NACRS = National Ambulatory Care Reporting System
NB = New Brunswick
NL = Newfoundland and Labrador
NS = Nova Scotia
ON = Ontario
pCMOD = pan-Canadian Minimal Oncology Dataset
PEI = Prince Edward Island
QB = Quebec
RCT = Randomized Clinical Trials
RWD = Real World Data
RWD = Real World Evidence
SK = Saskatchewan
WG = Working Group
